# Draft Genome Sequence of *Lactobacillus plantarum* Kanjika 2007, Isolated from Kanjika, a South Indian Traditional Food

**DOI:** 10.1128/genomeA.00924-16

**Published:** 2016-11-17

**Authors:** G. Divyashri, K. Rajagopal, S. G. Prapulla

**Affiliations:** a Microbiology and Fermentation Technology Department, CSIR-CFTRI, Mysore, India; b Department of Protein Chemistry and Technology, CSIR-CFTRI, Mysore, India; c Academy of Scientific and Industrial Research (AcSIR), New Delhi, India

## Abstract

The draft genome sequence of *Lactobacillus plantarum* Kanjika 2007, isolated from the South Indian staple, medicinal, and traditional food kanjika, is reported here. The whole genome consists of 3.16 Mb with a G+C content of 44.7% and 3,009 protein-coding genes, 78 tRNAs, and 4rRNAs (5S-23S-16S).

## GENOME ANNOUNCEMENT

*Lactobacillus* spp. are probiotic microbes that are generally recognized as safe and are used extensively in probiotic formulations*. Lactobacillus plantarum* Kanjika 2007, isolated from kanjika, a South Indian traditional food, has been found to possess many health benefits*.* As reported, this organism has been thought to possess antimicrobial activity and may be used for the commercial production of vitamin B_12_ (1). There are many more beneficial effects, but no reports of harmful ones, noted with this organism. In order to enhance the production of secondary metabolites, it is indispensable to understand the organism at the genome level, and hence we report here the whole-genome sequence of *L. plantarum* Kanjika 2007.

Illumina HiSeq 1000 and NextSeq 500 technologies (Illumina, Inc., USA) were used for whole-genome sequencing. A total of 5,618,892 paired-end reads of 151-nucleotide length were obtained. The NGS QC toolkit version 2.2.1 was used to process the data (removing low-quality bases and adapters; ≥70% of the reads ≥Q20). The processed reads were assembled with Velvet version 1.2.10, and scaffolding was performed using SSPACE version 3.0 and GapCloser version 1-12-r6. The final assembly contains 25 contigs, with a total size of 3,165,622 bp and an *N*_50_ contig length of 321.9 kb. The lengths of the contigs ranged from 530 bp to 803,921 bp, with a G+C mol% of 44.7%.

Gene prediction was performed by RAST (Rapid Annotations using Subsystem Technology) version 2.0. A total of 3,009 genes were predicted, along with 78 tRNAs and four rRNAs (5S-23S-16S). The draft genome sequence of *L. plantarum* Kanjika 2007 revealed the presence of vitamin B_12_ metabolic pathway genes and an antimicrobial peptide synthesis cluster. However, no cytotoxins were observed in the genome. The 16S rRNA sequence of this organism has been deposited under the accession number KF318862.1. The virulence coding regions, such as cytolysins and hemolysins, were not found. The genomic island was observed to contain bile salt hydrolase and lactose metabolic pathway genes (*lacABCDEF*). A tremendous amount of β-galactosidase activity was also noted.

### Accession number(s).

The sequences obtained in this study have been deposited in DDBJ/EMBL/GenBank under the accession number MJAM00000000.
